# The Churches' Opportunity: Hospital Sunday, 1917

**Published:** 1917-06-23

**Authors:** 


### The Hospital

The Workers' Newspaper of Administrative Medicine and Institutional Life, Administration, National Insurance and Health.

No. 1620, Vol. LXII. SATURDAY, JUNE 23, 1917. PRICE ONE PENNY

### THE CHURCHES' OPPORTUNITY.

### HOSPITAL SUNDAY, 1917.

The present are democratic times, and it is full of hopeful promise that an incumbent in the Diocese of London should have devoted personal service this year to help the man in the street to help the churches, to remove the impression which was spreading, that the churches of London are losing their interest in the great work done by the hospitals, and that their helping hand has been weakened. Experience proves, too, that the incumbent, recommends a remedy which, if applied by all the churches, would add increased support to the Hospital Sunday Fund, remove almost at once the impression that the Church is lagging behind in her obedience to the Divine command to heal the sick, and at the same time strengthen her hands amongst those whom she is seeking to win for Christ.

The plan followed is one suggested by The Hospital on more than one occasion in the past, and already adopted with success in parishes and cities where it has been tried under the direction of sympathetic persons with a gift for organisation. Each church forms a Hospital Committee, consisting of a chairman, honorary secretary, and treasurer, who constitute the permanent officers. They recruit a sufficient number of parishioners each year to form a band of workers who, under their direction, in accordance with rules approved by the vicar, arrange the plan and make throughout their parish the effort to raise an adequate sum for the hospitals in connection with the Hospital Sunday collections. The plan rests upon the knowledge that "the greater number of baptised "members of the Church do not attend a place of "worship on Hospital Sunday, and yet on the "whole are willing to give a contribution to the "hospitals if asked, which gives the Church her "opportunity to gather in the offerings of all her "children for the hospitals in London." In certain poor parishes where this method has been tried the total contribution of the church has been increased sometimes by 100 per cent.; sometimes by ten times its former yield to the Hospital Sunday Fund. The influence of the church and the parish too has been strengthened by bringing many people into sympathetic touch with the parish church, the ordinary collections have been substantially increased, the number of regular Church workers has grown, and the plan has been a public witness to every home in the parish that the Church is maintaining her position, as a leading influence with the neighbourhood for the support of the hospitals.

The appeal of the preachers in the churches throughout the Metropolis of the Empire on Hospital Sunday, June 24, rests this year upon facts so varied, convincing, and admittedly incontrovertible, as to make them irresistible to every patriotic man, woman, and child whom they may reach. Hospital Sunday falls this year at the end of the third year of the Great War, which has added materially to the cost of all essentials to efficient hospital maintenance, including food, physic, and supplies of every kind. In spite of this the Metropolitan hospitals have maintained their normal work for the sick poor, and have, in addition, by great devotion and enterprise made provision for the in-patient care, treatment, and expedited recovery of 150,000 wounded warriors, **the number of wounded of all Services so treated to date being equal to Three Complete Army Corps.** What member of the population is there who is not proud of the voluntary hospitals of the nation, a pride deepened and strengthened by the splendid services rendered during the war, which have caused them to occupy a principal position in the system whereby the enemy has been confounded, and will ultimately be overthrown?

The voluntary system has always stood for efficiency and personal service. Since the war both have progressed, for the voluntary hospitals have placed everything that is best in surgery, science, medicine, and nursing at the disposal of our wounded, whilst they have been and are responsible for training the surgeons, physicians, and nurses on service at the Front and at home. This is not the time to enlarge upon the bravery and devotion of our doctors, surgeons, practitioners, and nurses, many of whom have been faithful to their duty unto death. Their memory will endure in history, and be kept green and fresh in memorials preserved as sacred in our hospital chapels, colleges, and training schools where these noble men and women learnt to serve their country, and gladly to die for it. There has probably never been a time in the history of our Nation and Empire when the war strain has been even approximately as great as that, which has entailed the loss of many noble lives amongst members of the medical profession at home and abroad, through the self-denying devotion and sacrifice which they have continuously exhibited in the battle medicine and science have waged for the protection and recovery of our sailors and soldiers. None, but those constantly associated with the efforts put forth by members of the medical profession and the scientists, can have an idea of the valuable lives lost to our nation through continuous personal service on the part of some of the noblest and best doctors of our time. The truth is that the demand for men and women with technical knowledge and other gifts has been so continuously increasing as to make it more and more difficult, with the aid of everybody available, irrespective of age, to overtake and continuously to accomplish the work for others which is essential to our success in the war. Such work for the welfare and help of workers in the war, their wives and children, and those of our brave men fighting the enemy on land and sea has been, and is, paramount.

In face of the facts, have the Church and the Preachers ever before had a more heart-stirring and moving theme to enlighten and win the congregations on Hospital Sunday? We are a business nation, and it may be well further to show that not only the theme but the cause is worthy of the most liberal contributions of everybody it may reach. We have been looking through the administration and work done by the Metropolitan Hospital Sunday Fund in recent years. Sir Ernest Tritton's acceptance of the Vice-Presidency of the Metropolitan Hospital Sunday Fund and the changes which have taken place in the executive staff of the Fund in recent years have won the unanimous approval of the clergy, ministers, and men of business throughout London. Out of the volume of written testimony available the most striking and encouraging fact is the high appreciation won from everybody with knowledge by the present administrators and officers of the Fund. This appreciation testifies to "the consistent kindness received," "the good help for my parish," "the excellent administration of the Fund, and the absence of red tape," "the consideration shown to manifold requests," "gratitude for courteous and kindly help to parishioners and others," with the frequent statement that "our parish is continuously indebted to the Hospital Sunday Fund for valuable help." Add to all these encomiums the ever-increasing testimony on the part of individual clergymen and ministers to their **ambition to send a record amount, because out of their wide experience they have found "the Hospital Sunday Fund to be the best managed of all charities at the present time,"** and the evidence of splendid efficiency is complete indeed. We commend to each man who may have the privilege of preaching for Hospital Sunday in London this year a study of the pew slip, on the back of which he will find extracts from recent letters endorsing the brief summary we have made here of many gratifying and most notable facts.

